# Osteoporosis in Primary Care: An Analysis of Family Physicians' Knowledge, Attitudes, and Practices in Bahrain

**DOI:** 10.7759/cureus.79968

**Published:** 2025-03-03

**Authors:** Zahra S Alawi, Ebrahim Matar, Wafa F Hasan, Isa Y Althawadi, Aalaa Fakhrawi, Adla B Hassan, Mohamed H Shehata, Adel Alsayyad, Dalal A Al Hashel, Mahmood Al Saeed

**Affiliations:** 1 Internal Medicine, Salmaniya Medical Complex, Manama, BHR; 2 Public Health, Ministry of Health, Manama, BHR; 3 Internal Medicine, Middle East Hospital, Manama, BHR; 4 Endocrinology, King Fahad University Hospital, Khobar, SAU; 5 Internal Medicine, College of Medicine and Medical Sciences, Arabian Gulf University, Manama, BHR; 6 Family and Community Medicine, College of Medicine and Medical Sciences, Arabian Gulf University, Manama, BHR; 7 Family Medicine, Primary Health Care, Manama, BHR

**Keywords:** clinical knowledge, family medicine, fracture, knowledge attitude practices studies, osteoporosis

## Abstract

Background: Osteoporosis is a metabolic bone disease characterized by progressive loss of bone density. Osteoporotic fractures account for the significant morbidity and mortality of osteoporosis. Preventing and treating osteoporosis and its complications by proper screening and management will significantly improve prognosis and quality of life. Primary healthcare physicians play an important role in the primary prevention of chronic diseases. To that effect, we sought to assess the knowledge, attitude, and practices on the subject of osteoporosis among family physicians in Bahrain in order to identify the level of awareness in primary care centers.

Methodology: A cross-sectional study was conducted among family physicians working in primary healthcare centers across the Kingdom of Bahrain. Study participants were asked to complete an online revised osteoporosis knowledge test questionnaire. Data were collected and analyzed using IBM SPSS Statistics for Windows, Version 25 (Released 2017; IBM Corp., Armonk, New York).

Results: A total of 210 family physicians participated in this study. More than half of the participants had not received any structured education on osteoporosis or professional development sessions in the past five years (65.7%). Electronic resources were the most utilized type of resource for osteoporosis (73.8%, n=155). The overall mean score for knowledge was 57.2%. Participants strongly agreed that patient education is crucial for disease prevention (72.7%). The three questions that received the highest percentage of neutral responses were related to the ability to screen for osteoporosis in at-risk populations, the ability to correctly diagnose osteoporosis, and effectively manage a patient with osteoporosis in the clinic (34.3%, 36.7%, and 37.1%, respectively). When analyzing the data of practice questions, it was evident that the most used method for diagnosing osteoporosis among primary care physicians was the dual-energy X-ray absorptiometry scan (79%), while the least utilized method was the calculation of an osteoporosis score (14.8%).

Conclusion: Our research demonstrated average levels of knowledge on osteoporosis among family physicians in Bahrain. There is also a deficiency in receiving formal updated training. The results highlight several specific deficiencies in both osteoporosis-related knowledge and clinical practice. Therefore, we suggest the need for a well-developed national screening and awareness program to increase screening practices and enhance knowledge about osteoporosis in primary healthcare.

## Introduction

Osteoporosis is a metabolic bone disorder described as low bone mass with micro-architectural deterioration of bone tissue [1]. The main complication of osteoporosis is osteoporotic fractures, which account for this disease's morbidity and mortality [2]. In 2011, the International Osteoporosis Foundation reported that in nine industrialized countries in Europe, North America, Japan, and Australia, osteoporosis affected up to 49 million people [3]. Thirty percent of postmenopausal Caucasian women are estimated to have osteoporosis, according to the World Health Organization (WHO) criteria. Globally, nine million people suffer from fractures due to osteoporosis every year [3].

The impact of osteoporosis and its complications on healthcare costs can be quite immense [4]. One study in the United States reported that by 2025, fragility fractures will cost $25 billion annually [5]. In Europe, the annual cost of managing osteoporosis-related fractures is expected to increase to €76.7 billion by 2050 [6]. In Bahrain, osteoporosis is common, as reported by multiple prevalence studies. A study published in 2009 reported that the prevalence of osteoporosis in postmenopausal women was 27.1% [7]. Another retrospective study published in 2020 reviewed the bone mineral density (BMD) scan of a total of 205 Bahraini citizens [8]. This study showed that 38.0% of the participants were osteoporotic, while 46.5% were osteopenic [8]. Additionally, a cross-sectional study published in 2021 estimated the prevalence of low BMD among young Bahraini females and revealed that 34% of the participants had osteoporosis or osteopenia [9]. A recent multicenter Bahraini study in 2024 targeted a cohort of 4822 Bahraini subjects and revealed that the prevalence of low BMD was 62.3%, osteoporosis was 15.9%, and osteopenia was 46.4%; moreover, it showed that the highest prevalence of osteopenia was among younger adults [10]. The possible etiology for this high prevalence among Bahraini individuals could be multi-factorial, including lifestyle malpractices, vitamin D deficiency, and genetic factors, as has been shown among women from Saudi Arabia [11]. Osteoporosis is considered a silent disease with no associated warning signs prior to developing complications such as fractures. As a result, osteoporosis is usually underdiagnosed and undertreated in the general population [12].

Numerous guidelines have emerged advising on the screening practice for osteoporosis. One of the most commonly applied guidelines in the United States is the Preventive Service Task Force (USPSTF) guideline, which recommends screening for osteoporosis in postmenopausal women and elderly men [13]. Family physicians play a key role in osteoporosis management by conducting screening for early detection, implementing preventive strategies, and overseeing ongoing treatment and care [14]. Knowledge, attitude, and practice (KAP) surveys can assess the awareness about screening processes and resources that are essential for defining effective activities in the prevention and control of osteoporosis [14]. Studies based on KAP surveys may be used to assess needs, problems, and barriers in healthcare programs, as well as solutions to improve the quality and accessibility of services [14].

There is consistent evidence from various regional studies of the high incidence and prevalence of osteoporosis [10,15-19]. Investigating the KAP of physicians is recommended and assists in improving screening programs while adhering to national guidelines to screen for osteoporosis [19-21]. In 2013, a study in Abu Dhabi reported that more than 75% of local physicians were aware of the presence of regional guidelines on osteoporosis [19,22]. However, the study also demonstrated that the level of knowledge regarding several pharmacological treatment options was low [19,22]. Another study conducted in Saudi Arabia in 2014 identified a gap in physicians' knowledge regarding bone health and a gap in awareness about local and international osteoporosis guidelines [18]. In another Saudi study, investigators reported a significant association between the level of awareness and sociodemographic category of the participants, including age, gender, education, occupation, income, and area of residence [17]. A cross-sectional KAP study conducted in Al-Ahsa, Saudi Arabia, revealed a defect in knowledge about osteoporosis among family physicians [16]. In this particular study, researchers recommended developing educational and training programs for physicians in primary care centers to improve their approach toward osteoporosis [16].

In Bahrain, there is a deficiency in studies addressing osteoporosis practices. Thus, assessing the level of knowledge and awareness of osteoporosis is an important first step toward improving care, practice, and management. This would, in turn, contribute to the improvement of life quality and decreased disease burden. Therefore, the aim of the current study is to assess KAP related to osteoporosis among family physicians working at primary health centers in Bahrain and to identify the level of awareness in primary health centers. The findings will, in turn, contribute to the development of targeted policy recommendations and intervention strategies designed to enhance osteoporosis management, improve patient outcomes, and promote public awareness of bone health in Bahrain.

## Materials and methods

This cross-sectional, questionnaire-based study was conducted over three months (July 2023 to September 2023) in the private and public primary healthcare sectors in the Kingdom of Bahrain. All registered physicians providing primary care services, including family physicians, family medicine residents, and general practitioners, were eligible to participate. Physicians who were not actively practicing at the time of the study were excluded.

A soft copy of a self-administered questionnaire was distributed electronically to physicians to participate in the study, ensuring efficient and broad access among eligible participants. To enroll participants, a convenience sampling method was utilized. A formal invitation message accompanied the questionnaire link, outlining the study's purpose and providing clear instructions for completion. Most of the questions were adopted from a validated questionnaire developed by Mahdaviazad et al. with minor modifications [22]. The questionnaire is divided into four sections. The first section had questions about the demographic and professional qualifications, which included age, sex, employment location, nationality, qualification, years of practice, osteoporosis training courses, and the resources used for osteoporosis updates. The second section had 12 questions about the knowledge, which were scored using a three-point scale (yes, no, I don't know). The third section had six questions about the attitude, which were scored using a five-point scale (ranging from strongly agree to strongly disagree). The fourth section had six questions about the practice, which were answered by choosing from multiple choices or by answering in numbers. The questionnaire took approximately 10 minutes to complete. A pilot study was first conducted on 30 students to assess the validity and reliability of the questionnaire. The tool demonstrated an acceptable level of reliability, with a Cronbach's alpha of 0.76.

The study adhered to the Helsinki Declaration and received approval from the Research and Ethics Committee of Primary Healthcare in the Kingdom of Bahrain (approval number PHCRC/TOR/0014/2023) prior to data collection. Additionally, participants were provided with a study information sheet before the questionnaire, outlining details such as the study's objectives, the reasons for their selection, the benefits of participation, and assurances of confidentiality. Participants willing to participate were invited to provide informed consent before proceeding with the questionnaire.

Data were analyzed using the IBM SPSS Statistics for Windows, Version 25 (Released 2017; IBM Corp., Armonk, New York). Frequencies and percentages were used to describe categorical variables, while means and standard deviations were used for continuous variables. To assess the association between demographic and professional data with knowledge and attitude scores, the t-test was used for variables with large sample sizes per group (>30), while the Wilcoxon rank-sum test was used for variables with fewer observations per group (<30). A P-value < 0.05 was considered statistically significant.

## Results

In this study, 210 family physicians filled out the questionnaire assessing their knowledge, attitudes, and practices. Of the respondents, 169 (80.5%) were females, and 41 (19.5%) were males. Most of the participants were Bahraini nationals, 194 (92.4%). Regarding employment, 164 (78.1%) of the physicians served in the public sector, particularly in primary healthcare centers and the royal medical services, while only 46 (21.9%) physicians worked in the private sector. Most of the participants, 89.5% (189), were employed as full-time family physicians. More than half of the participants (65.7%, 138) did not receive any formal training on osteoporosis in the last five years. Electronic resources were the most utilized resource on osteoporosis (73.8%, 155), followed by lectures and symposiums (50%, 105). The least utilized resource was textbooks (23.8%, 50). The experience level varied among participants, with 62 (29.5%) practicing medicine for less than five years. This was followed by those with 5-10 years of practice (24.8%, 52), 11-20 years of experience (21.9%, 46), and more than 20 years (23.8%, 50). Regarding their professional ranking, many (37.1%, 78) were family medicine consultants (Table 1).

**Table 1:** Demographic and professional characteristics of the study participants FPRP: family practice residency program; SD: standard deviation

Sociodemographic Data of Participants			Count	Percentage
Gender		Female	169	80.5%
		Male	41	19.5%
		Total	210	100.0%
Age (mean ± SD)			40	11
Nationality		Non-Bahraini	16	7.6%
		Bahraini	194	92.4%
		Total	210	100.0%
Health center name		Public sector	164	78.1%
		Private sector	46	21.9%
		Total	210	100.0%
Work type		Part-time	22	10.5%
		Full-time	188	89.5%
		Total	210	100.0%
Professional grade		Family physician consultant	78	37.1%
		Family physician specialist	63	30.0%
		FPRP resident	22	10.5%
		GP	47	22.4%
		Total	210	100.0%
Number of years of practice		Less than 5 years	62	29.5%
		5 to 10 years	52	24.8%
		11 to 20 years	46	21.9%
		More than 20 years	50	23.8%
		Total	210	100.0%
Did you receive training on osteoporosis in the last five years?		No	138	65.7%
		Yes	72	34.3%
		Total	210	100.0%
What do you depend on as a resource for osteoporosis updates?	Textbooks	No	160	76.2%
		Yes	50	23.8%
		Total	210	100.0%
	Journals	No	153	72.9%
		Yes	57	27.1%
		Total	210	100.0%
	National guideline	No	123	58.6%
		Yes	87	41.4%
		Total	210	100.0%
	Electronic resources (Medscape, UpToDate, etc.)	No	55	26.2%
		Yes	155	73.8%
		Total	210	100.0%
	Lectures, symposiums	No	105	50.0%
		Yes	105	50.0%
		Total	210	100.0%

The overall mean score for the level of knowledge was 57.2%. The question with the highest number of correct answers was regarding whether using corticosteroids for more than three months is an indication for osteoporosis screening (86.7%), and the least question answered correctly was pertaining to the usage of bisphosphonates as the first-line treatment of osteoporosis (24.8%) (Table 2). Participants with more than 10 years of experience scored higher than those with less years of experience (59.4% vs. 55.2%; P = 0.043). Furthermore, the nationality of the practitioners was a significant factor, with non-Bahraini physicians scoring higher than Bahrainis (67.7% vs. 56.3%; P = 0.012) (Table 3).

**Table 2:** Responses of physicians to knowledge questions OP: osteoporosis; SD: standard deviation

Knowledge Questions		Count	Percentage
OP is the most common metabolic bone disease	No	18	8.6%
	Yes	160	76.2%
	Do not know	32	15.2%
	Total	210	100.0%
WHO criteria define a t-score equal to or less than -2.5 SD as OP	No	14	6.7%
	Yes	177	84.3%
	Do not know	19	9.0%
	Total	210	100.0%
WHO criteria define a T-score of -1 to -2.5 SD as osteopenia	No	16	7.6%
	Yes	176	83.8%
	Do not know	18	8.6%
	Total	210	100.0%
The process of bone loss starts around 40 years of age in both sexes	No	92	43.8%
	Yes	73	34.8%
	Do not know	45	21.4%
	Total	210	100.0%
Guidelines recommend screening average-risk postmenopausal women for osteoporosis beginning at age 60 years	No	71	33.8%
	Yes	109	51.9%
	Do not know	30	14.3%
	Total	210	100.0%
Corticosteroid therapy for more than three months is an indication for osteoporosis screening testing earlier than the standard screening age	No	9	4.3%
	Yes	182	86.7%
	Do not know	19	9.0%
	Total	210	100.0%
Wrist fractures occurring with a fall equivalent to standing height or less are an indication of osteoporosis screening	No	40	19.0%
	Yes	126	60.0%
	Do not know	44	21.0%
	Total	210	100.0%
BMD assessment by Quantitative calcaneal Ultrasound can be used to screen for osteoporosis	No	40	19.0%
	Yes	110	52.4%
	Do not know	60	28.6%
	Total	210	100.0%
Recommended calcium intake in OP is 800 mg/day	No	88	41.9%
	Yes	89	42.4%
	Do not know	33	15.7%
	Total	210	100.0%
All patients with OP should receive calcium supplements independent of dietary assessment	No	63	30.0%
	Yes	129	61.4%
	Do not know	18	8.6%
	Total	210	100.0%
Bisphosphonates are the first-line choice of OP treatment in both sexes	No	17	8.1%
	Yes	163	77.6%
	Do not know	30	14.3%
	Total	210	100.0%
Bisphosphonates are the first-line choice for OP prevention	No	110	52.4%
	Yes	52	24.8%
	Do not know	48	22.9%
	Total	210	100.0%

**Table 3:** Knowledge and attitude mean ± SD scores by demographic variables and professional data * The Wilcoxon rank-sum test was used to calculate p-values.

Variables		Knowledge Percent		Attitude Percent	
		Mean ± SD	P-value	Mean ± SD	P-value
Gender	Women	57.05 ± 14.37	0.830	75.01 ± 13.59	0.777
	Men	57.72 ± 18.66		74.31 ± 16.33	
Years of practice	≤10 years	55.26 ± 16.52	0.047	73.71 ± 13.97	0.195
	>10 years	59.46 ± 13.33		76.25 ± 14.26	
Health center type	Public	56.35 ± 13.53	0.235	74.33 ± 13.52	0.295
	Private	60.14 ± 20.17		76.81 ± 16.11	
Osteoporosis training course in the last five years	No	56.40 ± 16.12	0.307	74.32 ± 13.62	0.436
	Yes	58.68 ± 13.44		75.93 ± 15.08	
Nationality*	Non-Bahraini	67.70 ± 17.43	0.012	79.16 ± 14.38	0.146
	Bahraini	56.31 ± 14.78		74.52 ± 14.08	
Work type*	Part-time	59.47 ± 19.29	0.342	77.57 ± 15.87	0.253
	Full-time	56.91 ± 14.76		74.56 ± 13.92	

Regarding attitude, participants agreed strongly that patient education is crucial for disease prevention (72.7%). This was followed by the participants strongly agreeing that osteoporosis is a serious health concern that requires more attention in the practice of family physicians (65.7%). The top three questions receiving neutral responses were related to the ability to screen for osteoporosis in at-risk populations, correctly diagnosing osteoporosis, and effectively managing a patient with osteoporosis in their practice (34.3%, 36.7%, and 37.1%, respectively) (Table 4). Additionally, the mean attitude question scores showed no significant differences concerning demographic factors, type of practice, or type of practice (Table 3).

**Table 4:** Responses of physicians to attitude questions

Attitude Questions		Count	Percentage
Do you believe osteoporosis is a serious health concern that deserves more attention in family practice?	Strongly Disagree	11	5.2%
	Disagree	2	1.0%
	Neutral	18	8.6%
	Agree	41	19.5%
	Strongly Agree	138	65.7%
	Total	210	100.0%
Are you able to screen for osteoporosis in at-risk populations?	Strongly Disagree	22	10.5%
	Disagree	23	11.0%
	Neutral	72	34.3%
	Agree	46	21.9%
	Strongly Agree	47	22.4%
	Total	210	100.0%
Are you confident in your ability to correctly diagnose osteoporosis?	Strongly Disagree	10	4.8%
	Disagree	26	12.4%
	Neutral	77	36.7%
	Agree	56	26.7%
	Strongly Agree	41	19.5%
	Total	210	100.0%
Are you confident in managing a patient with osteoporosis in your practice?	Strongly Disagree	32	15.2%
	Disagree	48	22.9%
	Neutral	78	37.1%
	Agree	34	16.2%
	Strongly Agree	18	8.6%
	Total	210	100.0%
Pharmaceutical treatments are important in the management of osteoporosis.	Strongly Disagree	11	5.2%
	Disagree	6	2.9%
	Neutral	37	17.6%
	Agree	75	35.7%
	Strongly Agree	81	38.6%
	Total	210	100.0%
Patient education is crucial for disease prevention.	Strongly Disagree	10	4.8%
	Disagree	4	1.9%
	Neutral	8	3.8%
	Agree	35	16.7%
	Strongly Agree	152	72.7%
	Total	209	100.0%

Our results identified that the most used method for diagnosing osteoporosis among primary care physicians was the BMD or dual-energy X-ray absorptiometry (DEXA) scan (79%) (Table 5). This was followed by the assessment of medical history (66.7%). The least utilized method was the calculation of an osteoporosis score (14.8%). In terms of management approaches, the most frequently reported approach was advising for lifestyle changes, such as smoking cessation and fall prevention (86.7%), followed by the promotion of physical activity (77.1%). On the other hand, the least employed management approach was prescribing assistive devices to prevent falls (48.6%) (Table 5).

**Table 5:** Responses of physicians to practice questions BMD: bone mineral density; DEXA: dual-energy X-ray absorptiometry; FRAX: fracture risk assessment tool

Practice Questions			Count	Percentage
Which methods do you use to screen patients for osteoporosis?	Medical history	No	70	33.3%
		Yes	140	66.7%
		Do not know	0	0.0%
		Total	210	100.0%
	Physical examination	No	121	57.6%
		Yes	89	42.4%
		Do not know	0	0.0%
		Total	210	100.0%
	BMD/DEXA scan	No	44	21.0%
		Yes	166	79.0%
		Do not know	0	0.0%
		Total	210	100.0%
	X-ray	No	153	72.9%
		Yes	57	27.1%
		Do not know	0	0.0%
		Total	210	100.0%
	FRAX score calculation	No	172	81.9%
		Yes	38	18.1%
		Do not know	0	0.0%
		Total	210	100.0%
	Osteoporosis score calculation	No	179	85.2%
		Yes	31	14.8%
		Do not know	0	0.0%
		Total	210	100.0%
	Laboratory tests (including blood and urine tests)	No	155	73.8%
		Yes	55	26.2%
		Do not know	0	0.0%
		Total	210	100.0%
Which of the following do you use in the management of osteoporosis?	Prescription of pharmaceutical treatments (bisphosphonates, calcium supplements, vitamin D supplements, etc.)	No	59	28.1%
		Yes	151	71.9%
		Do not know	0	0.0%
		Total	210	100.0%
	Advising dietary changes	No	52	24.8%
		Yes	158	75.2%
		Do not know	0	0.0%
		Total	210	100.0%
	Advising physical activity	No	48	22.9%
		Yes	162	77.1%
		Do not know	0	0.0%
		Total	210	100.0%
	Advising lifestyle changes (smoking cessation, fall prevention)	No	28	13.3%
		Yes	182	86.7%
		Do not know	0	0.0%
		Total	210	100.0%
	Recommending assistive devices (to prevent falls)	No	108	51.4%
		Yes	102	48.6%
		Do not know	0	0.0%
		Total	210	100.0%
	Referral to orthopedics, endocrinologist	No	51	24.3%
		Yes	159	75.7%
		Do not know	0	0.0%
		Total	210	100.0%
	Other	No	209	99.5%
		Yes	1	0.5%
		Do not know	0	0.0%
		Total	210	100.0%

## Discussion

Osteoporosis is considered one of the most common metabolic bone diseases worldwide [3]. It can be a major cause of fractures and represents a significant health and economic burden. Preventing such complications may be achieved with the implementation of comprehensive assessments and screening regimens, which start at the primary healthcare level. To that effect, this cross-sectional study was conducted to assess the KAP of osteoporosis among 210 primary healthcare physicians in the Kingdom of Bahrain.

Our participants demonstrated a mean knowledge score of 57.2%. This is comparable to the findings of a study conducted in Egypt on secondary care professionals, where 58% of them scored a satisfactory knowledge level in domains on the risk of osteoporotic fractures and investigations of osteoporosis [23]. In a study conducted in Malaysia, only one-third of primary healthcare physicians achieved satisfactory performance in knowledge about osteoporosis [24].

The question least answered correctly in our study was about the usage of bisphosphonates as the first-line prevention of osteoporosis. This is consistent with other studies where more than half of the participants did not achieve a satisfactory score in questions on pharmacotherapy [23,24]. On the other hand, our findings were contrary to a study done in Kuwait in 2013 [15]. In this study, the majority of physicians correctly agreed that hormonal replacement therapy is effective in the prevention of osteoporosis in postmenopausal women (87.3%) [15].

When asked about recent osteoporosis training, only 34.3% of our study participants have received training on osteoporosis management within the last five years. This low percentage might be related to poor exposure to osteoporosis in the primary healthcare setting. Other reasons include time constraints, financial barriers, and a lack of institutional support. On the contrary, secondary care physicians have reported a higher percentage (66.4%) of those receiving training to access and apply guidelines for the management of osteoporosis in their daily practice [23].

A similar study done in Saudi Arabia revealed that age and gender had an insignificant association with knowledge of osteoporosis [16]. On the other hand, our study has shown that participants with more than 10 years of experience scored higher than those with less experience (59.4% vs. 55.2%, P = 0.043). This can be explained by the higher level of experience and acquired awareness regarding osteoporosis compared to the newly employed physicians. Similar results were also reported in a study done in Malaysia on primary healthcare physicians [25]. Alternatively, other studies conducted in Saudi Arabia and Spain demonstrated that lesser years of experience were significantly associated with a higher level of osteoporosis awareness [16,26].

Regarding attitude toward osteoporosis, most participants agreed that osteoporosis is a serious health concern that requires more attention in family practice (65.7%). This result is consistent with a study done in Riyadh, which revealed that 81% of the participants considered osteoporosis an important health problem [18]. This was also shown in studies done in the United Arab Emirates and Spain [19,26]. Less than half of the respondents in our study agreed that they can screen or diagnose osteoporosis effectively (44.3%, 46.2%), while only 24.8% agreed that they can manage osteoporosis effectively. This is inconsistent with the attitude of primary care physicians' perceptions reported in other studies, as reported by Chenot et al., revealing that 83% of physicians perceived themselves as competent in osteoporosis prevention and control [27]. A study conducted in Malaysia was also inconsistent with our findings, as almost half of the participants (44.6%) were confident in providing non-pharmacological treatment of osteoporosis, while 65% stated that family physicians should have access to prescribe bisphosphonates in primary healthcare [24]. This could be due to regional differences in training and exposure to osteoporosis cases in clinical practice.

Regarding the practice of participants in the present study, it was found that the commonly used method for diagnosing osteoporosis among primary care practitioners is the DEXA scan, which is used by 79% of health providers. Moreover, while DEXA scans are readily available in primary healthcare facilities in Bahrain, only 8% of the primary healthcare physicians in other primary healthcare facilities in the region, such as in Al-Ahsa, Saudi Arabia, had access to request DEXA scans [16]. Additionally, over 90% of Malaysian primary care physicians considered access to DEXA scans as one of the barriers to osteoporosis screening and management [24].

The second most common method used by the participants was medical history (66.7%). This is in agreement with a study done in Riyadh, which revealed that three-quarters of physicians often inquire about back pain and current cigarette smoking as part of their medical history [18]. In terms of management approaches, the most frequently reported approach in this study was advising for lifestyle changes, such as smoking cessation and fall prevention (86.7%), followed by promotion of physical activity (77.1%). These practices are similar to those of secondary care physicians in Egypt, where 55% stated that they always prescribe exercise programs for older patients [24]. Additionally, the majority of the participants do not use the FRAX score to screen for osteoporosis (81.9%). While the FRAX score is validated for use in the Bahraini population, there is a need for greater awareness and education among clinicians regarding its proper application. The lack of use may not be due to the score's inaccessibility but rather a gap in understanding when and how to use it effectively in clinical practice. Educating healthcare professionals and integrating the FRAX score into clinical workflows, potentially through automatic inclusion in reports, could improve its utilization.

Our study did have some limitations. Selection bias may have occurred as participation relied on self-enrollment by physicians, and the convenience sampling method was utilized. Additionally, the private sector was underrepresented due to challenges in reaching physicians in private hospitals. Despite these limitations, our study had notable strengths, including a high participation rate among family physicians in the public sector, encompassing primary care centers across the entire country. Furthermore, the study included a diverse range of participants, from experienced consultants to residents at the start of their training journey.

## Conclusions

Osteoporosis is a common metabolic illness globally, especially among older adults. Given the rise in the elderly population, the cases of osteoporosis are expected to increase. Our study demonstrated average levels of knowledge on the subject of osteoporosis among primary healthcare physicians in Bahrain. This could be due to inadequate formal specialized training on the subject and a lack of clinical exposure. A well-established national program, which includes screening, training, and management guidelines, is recommended in order to raise awareness and knowledge and empower healthcare professionals to identify osteoporosis cases and initiate treatment. Future studies should be conducted to evaluate the cost-effectiveness of screening programs for osteoporosis and the outcome of a structured health program to enhance knowledge, screening, and treatment practices among primary healthcare physicians in Bahrain.
